# Activity of Pemigatinib in Pilocytic Astrocytoma and *FGFR1*^*N546K*^ Mutation

**DOI:** 10.1200/PO.21.00371

**Published:** 2022-05-04

**Authors:** Stephen Capone, Leena Ketonen, Shiao-Pei Weathers, Vivek Subbiah

**Affiliations:** ^1^ Department of Neurosurgery, University of Texas MD Anderson Cancer Center, Houston, TX; ^2^ Department of Diagnostic Imaging, MD Anderson Cancer Center, Houston, TX; ^3^ Department of Neuro-Oncology, Division of Cancer Medicine, The University of Texas MD Anderson Cancer Center, Houston, TX; ^4^ Department of Investigational Cancer Therapeutics, Division of Cancer Medicine, The University of Texas MD Anderson Cancer Center, Houston, TX; ^5^ Division of Pediatrics, The University of Texas MD Anderson Cancer Center, Houston, TX; ^6^ MD Anderson Cancer Network, The University of Texas MD Anderson Cancer Center, Houston, TX

## Introduction

Pilocytic astrocytomas are primary CNS WHO grade I glial tumors and are the most common among childhood brain tumors.^1^ Although surgery remains as a primary treatment modality, novel nonsurgical options continue to be investigated as these tumors frequently harbor genomic alterations within the mitogen-activated protein kinase (MAPK) signaling pathway.^2,3^ Although the majority of pilocytic astrocytoma harbor an identified MAPK pathway alteration, in particular, *KIAA1549-BRAF* fusions, *BRAF*^*V600E*^ mutations, and *BRAF*^*insVLR*^, along with other *BRAF* fusions, a subset of these tumors are driven by alterations outside of these common drivers.^2,4-6^ Additional alterations identified by whole-genome sequencing with paired whole-transcriptome sequencing included fibroblast growth factor receptor 1 (*FGFR1*) mutations, *NTRK2* fusions, *NF1* mutations, and *PTPN11* mutations. Development of targeted treatments focused on these alterations and the MAPK pathway continues to be an area of interest in the treatment of glial tumors.

Here, we present a case of *FGFR1*^*N546K*^-mutated juvenile pilocytic astrocytoma successfully treated with a pan-*FGFR* inhibitor pemigatinib illustrating the intracranial activity.

## Case Report

A 32-year-old male patient presented to The University of Texas MD Anderson Cancer Center after treatment at an outside hospital to discuss additional advanced treatment options. The patient was diagnosed with juvenile pilocytic astrocytoma diagnosed at age 13 years. At that time, the patient presented with headache and double vision. On imaging, the patient was noted to have obstructive hydrocephalus because of a third ventricular lesion and underwent a bilateral ventriculoperitoneal shunt placement and biopsy of the mass. Pathology was diagnostic for pilocytic astrocytoma. The decision was made to monitor the patient with serial imaging, which showed interval progression at 4 months postbiopsy, after which the patient was referred to radiation oncology. That patient underwent hypothalamic intensity-modulated radiation therapy at a dose of 5,400 cGy in 30 fractions. The patient was followed with serial imaging for the next 11 years with no tumor progression noted, after which the patient left the country and was unable to obtain imaging. Several years later, the patient presented with worsening visual deficits described as a binasal hemianopia, imaging showed tumor progression of the third ventricular lesion, and the patient underwent a near gross total resection. Pathology at this time showed a recurrent pilocytic astrocytoma with anaplasia, noted to have Rosenthal fibers, granular bodies, and scattered mitotic figures. Immunohistochemical studies showed retained expression of *ATRX*, and few cells were positive for wild-type *p53* and negative for *BRAF*^*V600E*^ and histone H3K27M. Fluorescence in situ hybridization studies noted sporadic amplification of *PDGFR* in the form of double minutes. Additional studies showed no duplication or rearrangement of *BRAF* or deletion of *CDKN2A*. The patient's postoperative course was complicated by hypopituitarism and fluctuating sodium levels, including episodes of both hyponatremia and hypernatremia, ultimately leading to an admission to the intensive care unit. Once stabilized, additional radiation treatment was not considered because of potential cognitive side effects.

On presentation, the patient denied any neurologic or cognitive deficits but was found to be oriented only to person with a fund of knowledge that was impaired from that expected for his age and level of education. Physical examination was grossly unremarkable with cranial nerves II-XII intact, the muscle strength was 5/5 bilaterally in all extremities, all deep tendon reflexes were two bilaterally, and there were no abnormal reflexes (Babinski, Hoffman, and jaw jerk were all negative). The patient had no cerebellar symptoms including nystagmus, finger-to-nose test, heel-to-shin test, and tremors, and the patient's gait was normal.

**FIG 1.**
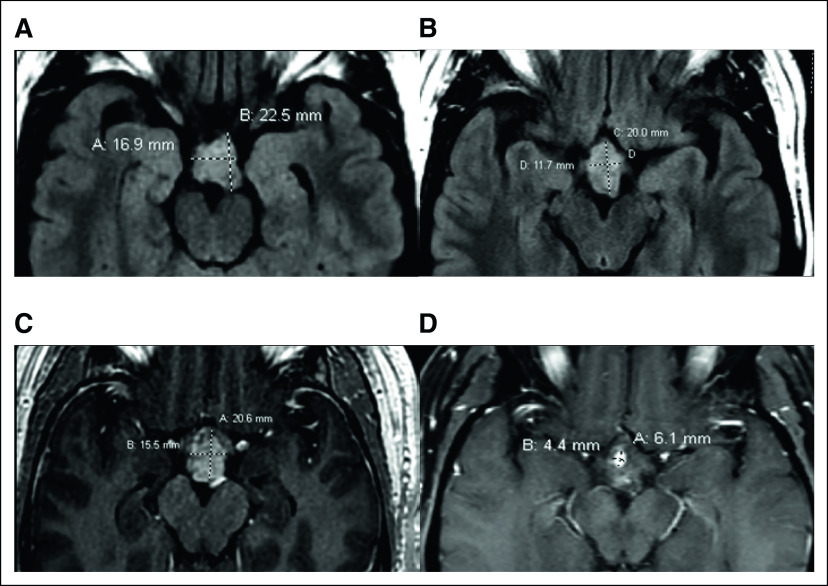
Baseline MRI images after intensity-modulated radiation therapy and near gross total resection of hypothalamic pilocytic astrocytoma. (A) Axial FLAIR and (B) postcontrast images demonstrating the residual enhancing tumor. After 10 months of treatment, hypothalamic tumor still demonstrates (C) good response with a significant reduction in tumor size in axial FLAIR and (D) only minimal residual focus of enhancement present in postcontrast MR images. MRI, magnetic resonance imaging.

Comprehensive next-generation sequencing was performed using the FoundationOne panel (Foundation Medicine; Cambridge, MA), which identified an *FGFR1*^*N546K*^ mutation (summarized in Table 1). Given his worsening deficits, after discussion with patient and consensus in the multidisciplinary tumor board, the patient was enrolled in an ongoing dose-escalation phase I/II clinical trial of pemigatinib in advanced malignancies with *FGFR* mutations (NCT02393248). After screening and baseline tests, the patient received 13.5 mg once daily oral dosing continuously on a 21-day cycle. Serial imaging was compared with the patient's baseline magnetic resonance imaging, and response was graded by RECIST V 1.1 (Figs 1A and 1B).^7^ The patient achieved a partial response with a 52% reduction at first restaging, which deepened to a 91% reduction as the best response in 13 cycles (Figs 1C and 1D) and sustained this response for a total of 18 months before a slight interval increase of the tumor on magnetic resonance imaging. The patient tolerated the treatment reasonably well for the first 6 months and had to undergo dose reduction to 9 mg once daily because of elevated liver function tests (grade 2 ALT and grade 1 AST from a history of fatty liver), mucositis (grade 1), and hand-foot syndrome (grade 2). Shortly thereafter, the imaging showed progressive disease approximately 19 months after treatment initiation. At this time, the decision was made to continue this course of treatment because of the continuous clinical benefit, despite radiographic progression up to 27 cycles at which time the patient was taken off the trial.

**TABLE 1.**
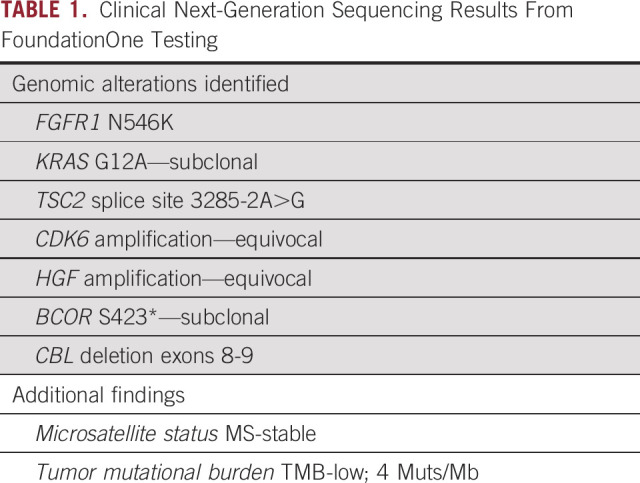
Clinical Next-Generation Sequencing Results From FoundationOne Testing

## Consent

Informed consent to publish information and/or images from the patient was obtained for this study.

## Discussion

Pemigatinib, a selective pan-fibroblast growth factor receptor (*FGFR*) inhibitor now US Food and Drug Administration–approved for use in locally advanced or metastatic cholangiocarcinoma with an *FGFR2* fusion or rearrangement, continues to be investigated in various neoplasms.^8-17^ This pan-*FGFR* inhibitor is a small-molecule kinase inhibitor that exerts its main effects in *FGFR1*, *FGFR2*, and *FGFR3* and a minor effect on *FGFR4* with a half maximal inhibitory concentration (IC50) of < 2 nM.^17-19^ *In vitro* preclinical data using cancer cell lines, including those from lung, gastric, endometrial, bladder, and hematologic malignancies, showed that pemigatinib can effectively inhibit phosphorylation of *FGFR1-3*, which decreased downstream signaling and cell viability.^18,19^ These lines all harbored various *FGFR* alterations, including amplifications, mutations, fusions, and translocations, all of which showed a response to pemigatinib.^18^ Related *FGFR* alterations in human cancers can lead to constitutive activation of the *FGFR* pathway, leading to increased survival and malignant transformation. *In vivo* studies using a mouse xenograft model implanted with *FGFR1-3*–altered human tumors all showed antitumor activity of pemigatinib, including models of cholangiocarcinoma expressing the *FGFR2-Transformer-2 beta* homolog (*TRA2b*) fusion protein, *FGFR2*-amplified gastric cancer*, FGFROP2-FGFR2* fusion–positive leukemia, and *FGFR3-TACC* fusion bladder carcinoma.^18,19^ Taken in combination, the preclinical in vitro and in vivo data show efficacy of pemigatinib across a wide variety of alterations within the *FGFR* pathway.

**FIG 2.**
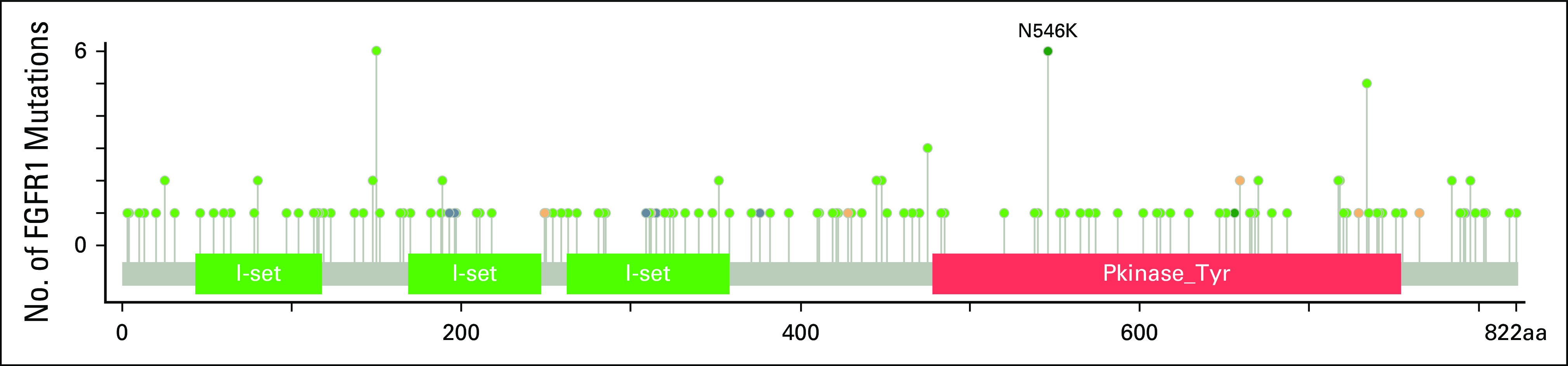
Lollipop figure of FGFR1 from cBioPortal showing *FGFR1*^*N546K*^ mutation in the tyrosine kinase domain.

*FGFR1*^*N546K*^ is a hot spot mutation in the tyrosine kinase domain (Fig 2), known to be activating and oncogenic, and is predominantly seen in CNS tumors.^20,21^ This mutation resides within the kinase binding domain of the *FGFR1* gene, unlike other oncogenic mutations within *FGFR1*, and does not appear to alter the tertiary structure of the protein, but does alter the surface charge.^22^ The *N546K* mutation has been implicated in the in vitro transformation of cells and has shown altered autophosphorylation, leading to increased catalytic activity and downstream activation of the MAPK pathway.^2,23^ *FGFR1*^*N546K*^ is present in 0.11% of AACR GENIE cases, with low-grade glioma not otherwise specified (NOS), conventional glioblastoma multiforme, glioblastoma, high-grade glioma NOS, and rosette-forming glioneuronal tumor of the fourth ventricle having the greatest prevalence (Dataset v10.0, available via AACR Project GENIE cBioPortal).^24,25^ Although it shows the prevalence of this mutation within CNS tumors, this data set does not represent the totality of the genetic landscape.^24^ Intriguingly, *FGFR1*^*N546K*^ has been described as a resistance mutation to four ATP-competitive inhibitors: ponatinib, dovitinib, PD173074, and BGJ-398.^26^ Intracranial activity of pemigatinib shows that not only it is able to cross the blood-brain barrier, but also it is able to do so at a concentration that preserves its efficacy and inhibition of the *FGFR* pathway. Subsequent dose reductions because of toxicity could have led to loss of efficacy in addition to contribution by co-occurring alterations, in particular, KRAS^*G12A*^. This mutation although subclonal is downstream of the FGFR1^*N546K*^ mutation and might have contributed to the resistance to pemigatinib. Although this is not an actionable alteration at this time, it highlights the need to continue developing additional targeted therapies and to comprehensively characterize the molecular alterations in primary tumors and track alterations that arise or become clonally dominant throughout treatment and recurrence.

This is of particular interest in pilocytic astrocytoma, as *FGFR* alterations are well-established drivers in a subset of patients.^2,3,27^ Although not the most common drivers of disease, this subset of tumors lend themselves to systemic treatment if not able to be surgically cured. Interestingly, this cohort showed that all *FGFR1*-mutated tumors were extracerebellar and commonly appeared in midline locations.^2^ These tumors that arise in deep midline locations are challenging surgical candidates, with significant difficulties in accessing the lesion and achieving a gross total resection. The opportunity to avoid surgery in a subset of patients or to treat postoperative patients with residual tumor using adjuvant pemigatinib has the potential to decrease the associated morbidity of these tumors.^28^ These deep midline lesions tend to have decreased progression-free survival and increased rates of visual deficits, endocrine dysfunction, hearing abnormalities, and cranial nerve deficits.^28^

This observed intracranial and preserved antitumor activity in a glial tumor suggests that pemigatinib and other pan-*FGFR* inhibitors should be explored in higher-grade gliomas, including anaplastic astrocytoma, glioblastoma, and gliosarcomas, all of which have few treatment options at this time. Of note, glioblastoma multiforme has been shown to also harbor *FGFR*-driving alterations in both adult and pediatric patients,^29^ including a relatively high prevalence of *FGFR-TACC* fusions.^30,31^ This *FGFR-TACC* fusion has also shown sensitivity to *FGFR* inhibition,^31^ and as previously shown, pemigatinib has in vitro antitumor efficacy when targeting these fusion proteins.^18^

This signal of activity and duration warrants a prospective study assessing the use of pemigatinib and other pan-*FGFR* inhibitors in primary CNS tumors with an underlying *FGFR* alteration as monotherapy and in combination.
